# The Effect and Mechanism of KLF7 in the TLR4/NF-*κ*B/IL-6 Inflammatory Signal Pathway of Adipocytes

**DOI:** 10.1155/2018/1756494

**Published:** 2018-11-26

**Authors:** Meixiu Zhang, Cuizhe Wang, Jinxiu Wu, Xiaodan Ha, Yuchun Deng, Xueting Zhang, Jingzhou Wang, Keru Chen, Jiale Feng, Jiaojiao Zhu, Jianxin Xie, Jun Zhang

**Affiliations:** Laboratory of Xinjiang Endemic and Ethnic Diseases, Shihezi University School of Medicine, Bei-Er-Lu, Shihezi, Xinjiang 832000, China

## Abstract

**Objective:**

To investigate the role and possible molecular mechanism of Krüppel-like factor 7 (KLF7) in the TLR4/NF-*κ*B/IL-6 inflammatory signaling pathway activated by free fatty acids (FFA).

**Methods:**

The mRNA and protein expression levels of KLF7 and the factors of TLR4/NF-*κ*B/IL-6 inflammatory signal pathways were detected by qRT-PCR and Western blotting after cell culture with different concentrations of palmitic acid (PA). The expression of KLF7 or TLR4 in adipocytes was upregulated or downregulated; after that, the mRNA and protein expression levels of these key factors were detected. KLF7 expression was downregulated while PA stimulated adipocytes, and then the mRNA and protein expressions of KLF7/p65 and downstream inflammatory cytokine IL-6 were detected. The luciferase reporter assay was used to determine whether KLF7 had a transcriptional activation effect on IL-6.

**Results:**

(1) High concentration of PA can promote the expression of TLR4, KLF7, and IL-6 in adipocytes. (2) TLR4 positively regulates KLF7 expression in adipocytes. (3) KLF7 positively regulates IL-6 expression in adipocytes. (4) PA promotes IL-6 expression via KLF7 in adipocytes. (5) KLF7 has a transcriptional activation on IL-6.

**Conclusion:**

PA promotes the expression of the inflammatory cytokine IL-6 by activating the TLR4/KLF7/NF-*κ*B inflammatory signaling pathway. In addition, KLF7 may directly bind to the IL-6 promoter region and thus activate IL-6.

## 1. Introduction

Obesity has been reported to be a chronic low-inflammation state. The release of adipocyte inflammatory factors is closely related to the increase of blood free fatty acid levels caused by obesity [1, 2]. The inflammation caused by obesity, which further induces insulin resistance, is the key factor to the occurrence of metabolic syndrome [3, 4].

Toll-like receptors (TLRs) play an important role in the inflammatory signaling pathway of the innate immune response [5]. Studies have shown that high levels of free fatty acids in the obese state induce immune inflammatory responses by binding to the adipocyte membrane receptor TLR4 and induce the expression and secretion of inflammatory-related factors, such as tumor necrosis factor alpha (TNF-*α*) and interleukin-6 (IL-6) [6–12].

Krüppel-like factors (KLFs) are a class of transcription factors with zinc finger structures. The KLF family has 17 members that are widely involved in the regulation of multiple biological events, such as cell proliferation, differentiation, and embryonic development [14–16]. KLF7 is localized in the nucleus and plays a role in transcriptional activation [16, 17]. In mature adipocytes, KLF7 regulates the expression of a number of adipogenesis-related genes and the adipocytokine, such as inhibiting the expressions of adiponectin and leptin [18–21]. The above-cited reports indicate that KLF7 may be involved in regulating the lipid metabolism and endocrine function of adipocytes. There are few researches on KLF7 in the obesity-induced inflammation. The IL-6 expression can be promoted by upregulating the KLF7 expression in human preadipocytes [19]. In our previous study, TLR4/KLF7/IL-6 was overexpressed in adipose tissue of obese adults and obese rats induced by a high-fat diet [22]. Moreover, there was a significant positive correlation between the TLR4 and the KLF7 expression and a positive correlation between KLF7 and both LDL and IL-6 [22]. The above-cited study and previous results suggest that KLF7 may be closely related to IL-6 overexpression induced by high levels of free fatty acids in obesity.

In our present study, we want to investigate the role of KLF7 in the process of FFA-induced IL-6 overexpression and to elucidate its possible mechanism in adipocytes. A new experimental basis is provided to clarify the molecular mechanism of FFA-induced adipocyte inflammatory response.

## 2. Methods

### 2.1. Cell Culture and Adipocyte Differentiation

3T3-L1 cells were cultured and differentiated into mature adipocytes in six-well plates. The culture medium consisted of Dulbecco's modified Eagle's medium (DMEM), 10% fetal bovine serum (FBS), and 1% penicillin-streptomycin solution. When the cell coverage reached 100%, the cells were differentiated for 2 days to produce Liquid 1, which contained DMEM, 10% FBS, 0.5 mmol/L 3-isobutyi-1-methyixanthine (IBMX), 1 *μ*M/L dexamethasone (DXMS), and 10 *μ*g/mL insulin. Then, the medium was changed to induce Liquid 2 for 2 days. The induced Liquid 2 contained DMEM, 10% FBS, and 10 *μ*g/mL insulin. Cells were further cultured in DMEM and 10% FBS for 4 days in the last step of induction. The differentiated adipocytes were identified by Oil Red O staining after 8 or more days of 3T3-L1 differentiation. The accumulation of large lipid droplets was observed. The induction rate reached 90%–100%, which was suitable for the experiment. Then, we started the transfection and stimulation experiment.

### 2.2. Preparation of Palmitic Acid Solution

Palmitic acid (0.0307 g) was added to 3 mL of 0.1 mol/L NaOH solution, which was then placed in a 75°C full saponification water bath for about 30 min until the liquid became colorless and transparent. Immediately thereafter, the palmitic acid solution was quickly added to 3 mL of 40% BSA (free of fatty acid) solution. The resultant solution was then mixed, filtered, and stored at −20°C. In our present study, adipocytes were stimulated with different concentrations of PA (0, 0.2, 2, 20, 100, and 200 *μ*mol/L) for 48 h.

### 2.3. Plasmid Construction and Transfection

The overexpression plasmid and the small interfering RNA (siRNA) interference fragment of TLR4 and KLF7 were constructed by GenePharma company. The overexpression plasmid of mouse TLR4 is pcDNA3.1(+)TLR4. Its negative control plasmid was pcDNA3.1(+)NC, which does not contain the promoter region of the TLR4 gene. The overexpression plasmid of human KLF7 was pcDNA3.1(+)KLF7. Its negative control plasmid was pcDNA3.1(+)NC, which does not contain the promoter region of the KLF7 gene. The overexpression plasmid of mouse KLF7 is pEX-3. Its negative control plasmid is pEX-3 (NC), which does not contain the promoter region of the KLF7 gene. The siRNA sequence is shown in Table 1. The plasmid or siRNA was transfected into adipocytes for 24 or 48 h by using Lipofectamine 2000. The luciferase plasmid pGL-3-Basic 2001 was constructed from upstream 1901 bp of the human IL-6 gene transcription start site to downstream 100 bp of the gene transcription start site (for a total length of 2001 bp). The promoter region of the human IL-6 gene was included in this fragment. Luciferase plasmid pGL-3-Basic splicing variants 1501, 1001, and 501 and the human KLF7 overexpression plasmid (GenePharma) were constructed. At the same time, PRL-TK Renilla luciferase plasmid (GenePharma) was purchased. The plasmids were transfected into 293T cells by Lipofectamine 2000.

### 2.4. Quantitative Real-Time Polymerase Chain Reaction

Cells were lysed with TRIzol reagent, and the total RNA was extracted. The RNA purity was determined by using an Agilent 2100 Bioanalyzer (Agilent Technologies, Santa Clara, CA, USA). The amount of RNA used to reverse transcribe 20 *μ*L of cDNA was 1 *μ*g. The reverse transcription (Eppendorf AG, Germany) program settings were 42°C for 60 minutes and 70°C for 15 min. The mRNA expression was detected by using a qRT-PCR instrument (QIAGEN, Hilden, Germany) with the following program settings: 95°C for 2 min, 40 cycles at 95°C for 5 seconds, and 60°C for 34 seconds. *β*-Actin was used as internal control. Data were obtained as Ct values, and the 2^−ΔCt^ method was used in the analysis. Gene expression was quantified using a relative method.  Table 2 shows the primer sequences used.

### 2.5. Western Blot

Protein was lysed by using lysis buffer containing 1% PMSF in RIPA to extract the total cellular protein. Then, the protein concentration was tested and adjusted. The 4 × SDS-PAGE loading buffer added to each sample was one-third of the protein volume. The protein was heated at 100°C for 10 min. The GAPDH protein expression was used as an internal control. Membranes were incubated at 4°C overnight with antibodies to anti-GAPDH (36 kDa; Zhongshan Jinqiao, China), TLR4 (97 kDa; Abcam), KLF7 (25 kDa; Abcam), IL-6 (19 kDa, Cell Signaling Technology), p65 (60 kDa, Abcam), and pp65 (s536, 65 kDa, Cell Signaling Technology) at a working ratio of 1 : 1000. A second antibody was incubated at room temperature for 2 h at a working ratio of 1 : 10,000. The chemiluminescence reagent (SuperSignal West Femto Maximum Sensitivity Substrate; Thermo Scientific) was used and detected with a ChemiScope mini-imaging system.

### 2.6. Luciferase Reporter Assay

The *IL-6* promoter region gene sequence was searched in the NCBI database, and the JASPAR database was used to predict the binding sites of the KLF7 and *IL-6* promoter regions. The luciferase plasmid (2001 bp) and its splicing variant luciferase reporter gene plasmid (1501 bp, 1001 bp, and 501 bp), the KLF7 overexpression plasmid, and the PRL-TK Renilla luciferase plasmid were transfected into 293 cells. The equipment used for detection included a full wavelength scanning microplate reader (BioTeK) and the Dual-Glo® luciferase assay system (E2920; Promega). The sample loading test applied the following steps: (1) add Dual-Glo® luciferase reagent to the plate, (2) incubate at 20–25°C for 10 minutes, (3) measure the firefly luminescence, (4) add Dual-Glo® Stop and Glo® reagent to the plate, (5) incubate at 20–25°C for 10 minutes, (6) measure the Renilla luminescence, (7) calculate the ratio of firefly/Renilla luminescence for each well, and (8) normalize the ratio in the sample well to that in a control well (or a series of control wells).

### 2.7. Data Analysis

The SPSS version 17.0 software package (SPSS Inc., Chicago, IL, USA) was used in the statistical analysis. The data for each group is derived from three replicates of the in vitro experiment. When the data is in a normal distribution, differences among groups were evaluated, using the Student *T* test of two independent samples and one-way ANOVA of three or more independent samples. When the data does not conform to the normal distribution, differences among groups were evaluated using the Kruskal-Wallis *H* test to perform statistical analysis on data with three or more independent samples and Wilcoxon rank sum test to perform statistical analysis on data with two independent samples. A *P* value of <0.05 was considered statistically significant.

## 3. Results

### 3.1. High Levels of Palmitic Acid Can Promote TLR4, KLF7, and IL-6 Expression in Adipocytes

**Figure 1:**
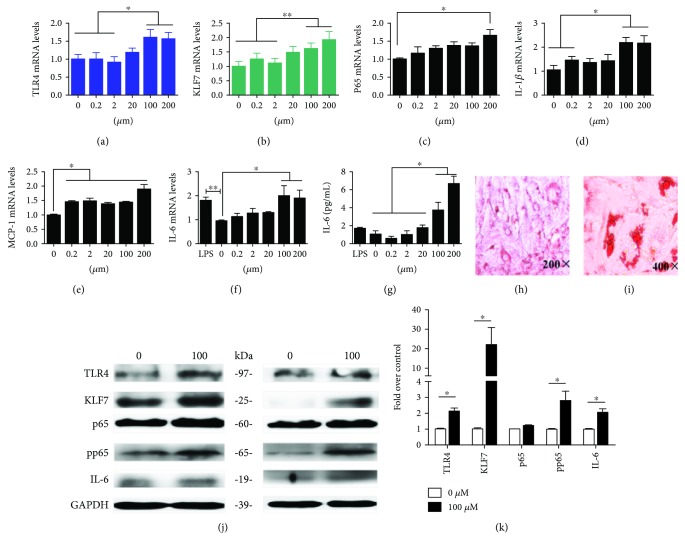
The mRNA expressions of TLR4, KLF7, and inflammatory cytokines (a–f) in adipocytes and the IL-6 protein release level (g), which were treated with 0, 0.2, 2, 20, 100, and 200 *μ*mol/L PA. The protein expression of TLR4, KLF7, and inflammatory cytokines in adipocytes was stimulated by 100 *μ*mol/L PA (j). Differentiation of 3T3-L1 preadipocytes into mature adipocytes, and Oil Red O staining of lipid droplets as viewed under a microscope at (h) 200x and (i) 400x magnification. Grayscale scanning of proteins (k). The data for each group is derived from three replicates of experimental data. One-way ANOVA (a, c–g), Kruskal-Wallis *H* test (b), *T* test (k), ^∗^*P* < 0.05; the difference was statistically significant.

After 8 days of 3T3-L1 induction, a large number of lipid droplets were observed under the microscope after Oil Red O staining (Figures 1(h) and 1(i)). The induction rate reached 90%–100%, which was suitable for the experiment.

Adipocytes were stimulated with different concentrations of palmitic acid (PA) for 48 h, and the expression of TLR4, KLF7, and IL-6 was detected. The results showed that low concentrations of PA (0, 2, and 20 *μ*mol/L) had no significant effect on the expression of these factors in adipocytes. However, stimulated by 100 and 200 *μ*mol/L PA, the mRNA expressions of TLR4, KLF7, nuclear factor-*κ*B protein p65 (p65), IL-6, interleukin-1*β* (IL-1β), and monocyte chemotactic protein 1 (MCP-1) were significantly higher than those in the control group, among which the IL-6 expression was more significantly increased (Figures 1(a)–1(f)). The TLR4, KLF7, p65, phosphorylation p65 (pp65), and IL-6 protein expression levels were significantly increased after 48 h of stimulation with 100 *μ*mol/L PA (Figures 1(j) and 1(k)). These results suggest that high concentrations of PA promote the expression of KLF7 and TLR4/NF-*κ*B and further increase the expression of IL-6 and other downstream inflammatory factors.

### 3.2. TLR4 Positively Regulates KLF7 and pp65 Expression in Adipocytes

**Figure 2:**
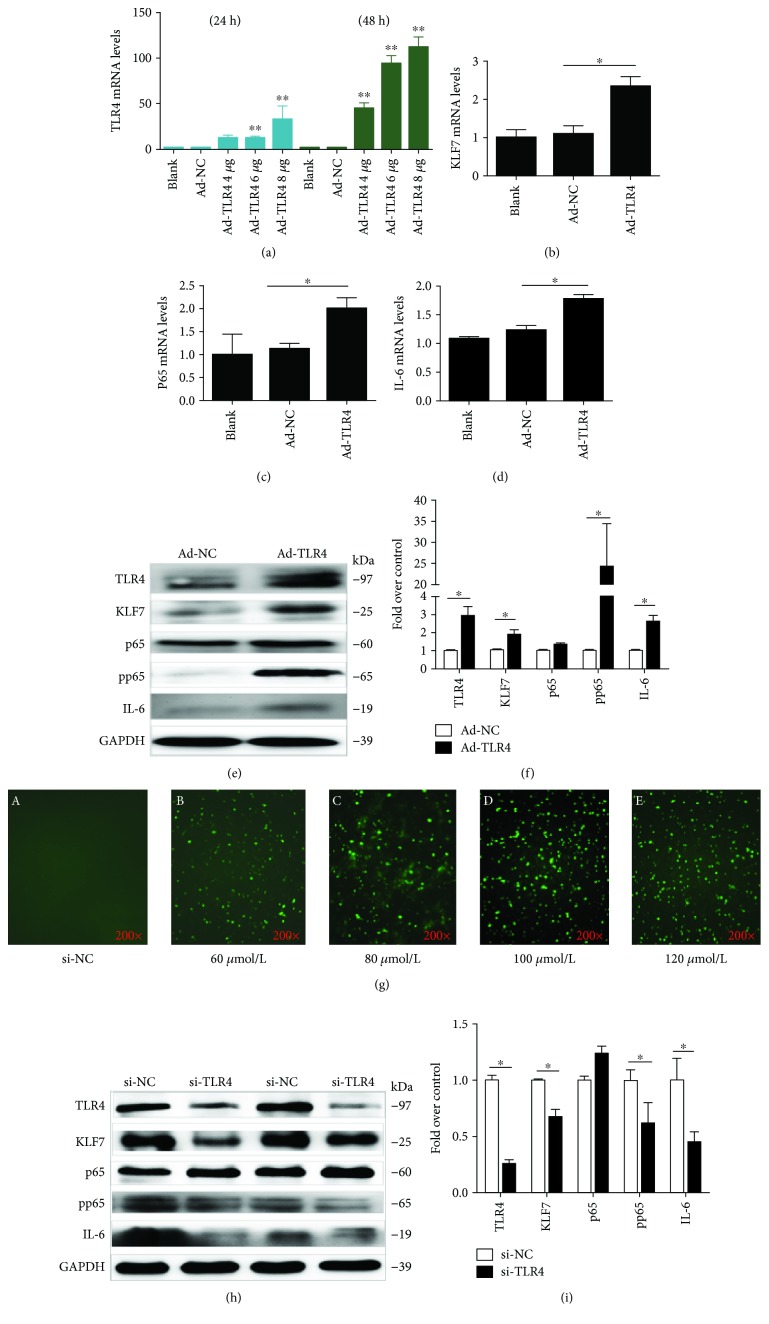
The TLR4 expression level in cells after 24 h and 48 h upregulation of the TLR4 overexpression plasmid (a). The mRNA expression of KLF7, p65, and IL-6 in adipocytes was detected after 48 h of upregulation of the TLR4 expression (b–d). The protein expression levels of KLF7, pp65, and IL-6 in adipocytes after 48 h upregulation of the TLR4 expression (e). The transfection efficiency after 8 h transfection with different concentrations of si-FAM fluorescent fragments into adipocytes (g). The protein expression levels of KLF7, pp65, and IL-6 in adipocytes after 48 h downregulation of the TLR4 expression (h). Grayscale scanning of proteins from (e) and (h) (f, i) (Ad-TLR4: transfected TLR4 overexpression plasmid, Ad-NC: transfected TLR4 negative control overexpression plasmid, siTLR4: transfected siRNA of TLR4, and siNC: transfected negative control of siRNA). The data for each group is derived from three replicates of experimental data. One-way ANOVA (a–d), *T* test (f, i), ^∗^*P* < 0.05, ^∗∗^*P* < 0.01; the difference was statistically significant.

After transfecting into adipocytes with different concentrations of TLR4 overexpression plasmids (4, 6, and 8 *μ*g/mL) for 24 h and 48 h, the TLR4 mRNA expression levels were significantly increased (*P* < 0.01; Figure 2(a)); the highest efficiency was achieved with a concentration of 8 *μ*g/mL for 48 h (*P* < 0.001). The mRNA and protein expressions of KLF7, pp65, and IL-6 were significantly increased after transfecting 8 *μ*g/mL TLR4 overexpression plasmids into adipocytes for 48 h (*P* < 0.05; Figures 2(b)–2(f)).

After 8 h transfection of adipocytes with different concentrations of si-FAM, fluorescent fragments were observed under a microscope, and the best transfection efficiency was found at 100 *μ*M/L (Figure 2(g)). After transfecting 100 *μ*mol/L TLR4 siRNA into adipocytes for 48 h, the protein expression of KLF7 was significantly decreased, as well as the protein expression levels of pp65 and IL-6 (Figures 2(h) and 2(i)). There was no significant change in the protein expression level of p65.

### 3.3. KLF7 Positively Regulates IL-6 and pp65 Expression in Adipocytes

**Figure 3:**
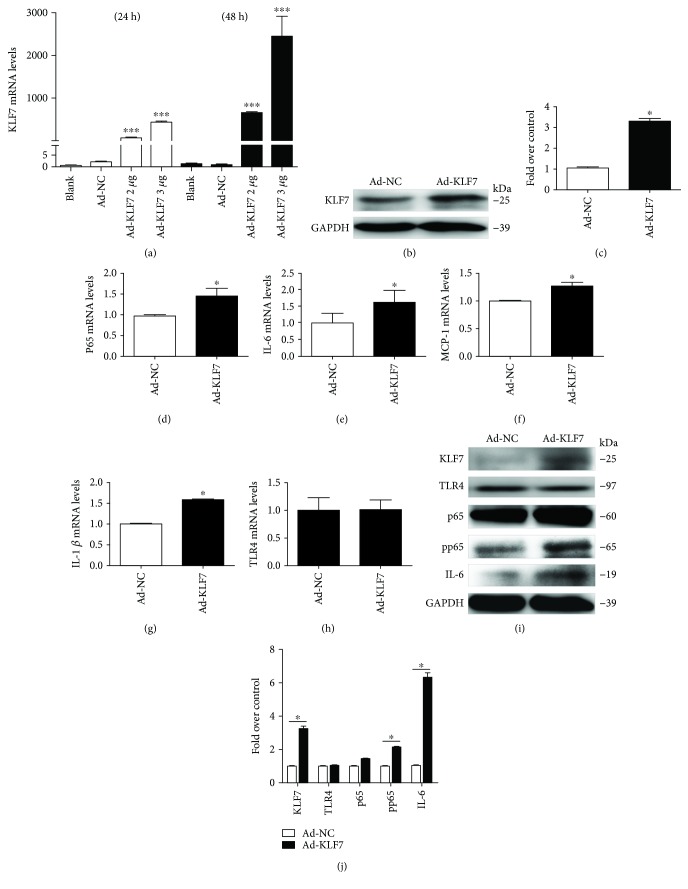
Detection of KLF7 mRNA expression at 24 h and 48 h after transfection of mature adipocytes with 3 *μ*g/mL KLF7 overexpression plasmid (a). The expression of KLF7 protein was further detected after 48 h of transfection (b, c). The mRNA and protein expression of inflammatory factors in adipocytes after 48 h transfection with KLF7 overexpression plasmids (d–i). Grayscale scanning of (b) and (i) proteins (c, j) (Ad-KLF7: transfected KLF7 overexpression plasmid; Ad-NC: transfected KLF7 negative control overexpression plasmid). The data for each group is derived from three replicates of experimental data. One-way ANOVA (a), *T* test (c, d, f–h, j), Wilcoxon rank sum test (e), Ad-NC: Ad-KLF7, ^∗^*P* < 0.05, ^∗∗∗^*P* < 0.001; the difference was statistically significant.

After the upregulation of the KLF7 expression in adipocytes by using the KLF7 overexpression plasmid (2 and 3 *μ*g/mL), the KLF7 mRNA expression level was significantly increased at 24 h and 48 h. The efficiency of KLF7 upregulation was highest at 48 h after transfection with 3 *μ*g/mL KLF7 overexpression plasmid (Figure 3(a)), and the KLF7 protein expression was significantly increased under this condition (Figures 3(b) and 3(c)). The mRNA expression levels of p65, IL-6, and MCP-1 were significantly increased after 3 *μ*g/mL KLF7 overexpression plasmid was transfected into adipocytes for 48 h (*P* < 0.05; Figures 3(d)–3(g)). At the same time, the p65, pp65, and IL-6 protein expression levels increased significantly (Figures 3(i) and 3(j)). However, there was no significant change in the expression level of TLR4 after the KLF7 overexpression (Figures 3(h) and 3(i)).

**Figure 4:**
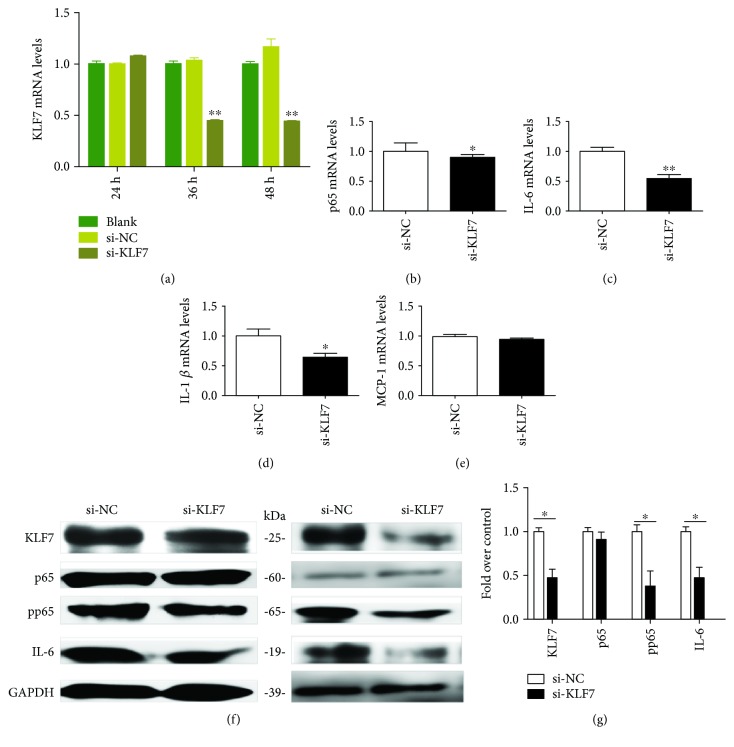
The KLF7 mRNA expression level in adipocytes was measured after KLF7 was downregulated for 24 h, 36 h, and 48 h, with 100 *μ*mol/L as the optimal concentration (a). After 48 h of KLF7 downregulation, the mRNA and protein expressions of inflammatory cytokines in adipocytes were measured (b–f). Grayscale scanning of proteins (g) (siKLF7: transfected siRNA of KLF7; siNC: transfected negative control of siRNA). The data for each group is derived from three replicates of experimental data. One-way ANOVA (a), *T* test (b–e, g), ^∗^*P* < 0.05, ^∗∗^*P* < 0.01; the difference was statistically significant.

The KLF7 mRNA expression level was significantly lower than those in the si-NC group at 36 h and 48 h after transfection with 100 *μ*mol/L KLF7 siRNA in mature adipocytes. The highest downregulation efficiency was achieved after 48 h (*P* < 0.01; Figure 4(a)). The downregulation of KLF7 by adipocytes transfected with 100 *μ*mol/L KLF7 siRNA for 48 h significantly decreased the expression of the downstream inflammatory factors p65, IL-6, and IL-1*β* (*P* < 0.05; Figures 4(b)–4(e)). The pp65 and IL-6 protein expression levels were also significantly decreased (Figures 4(f) and 4(g)).

### 3.4. PA Promotes IL-6 Expression via KLF7 in Adipocytes

**Figure 5:**
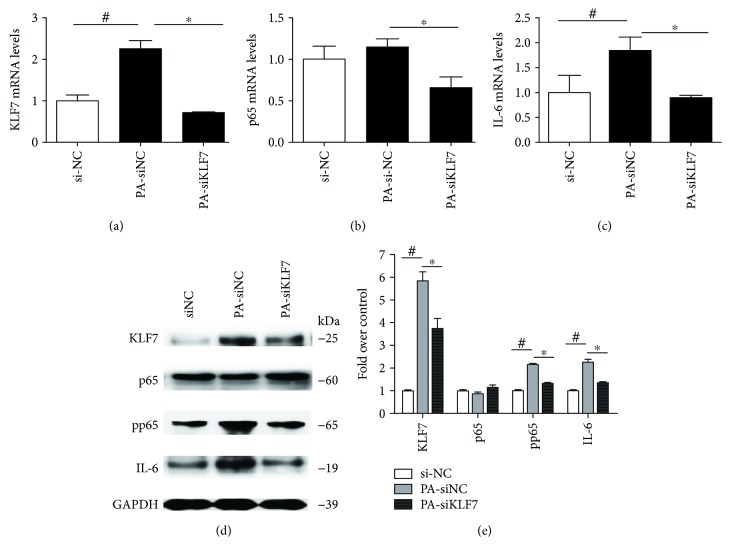
The mRNA (a–c) and protein (d, e) expression levels of inflammatory cytokines in adipocytes were detected by using PA to stimulate adipocytes and downregulate the KLF7 expression for 48 h. Grayscale scanning of proteins (e) (siNC: negative control of siRNA, PA-siNC: PA and negative control of siRNA, PA-siKLF7: PA and siKLF7). The mRNA data for each group is derived from three replicates of experimental data. One-way ANOVA (a–c, e), PA-siNC: PA-siKLF7, ^∗^*P* < 0.05; siNC: PA-siNC, ^#^*P* < 0.05. The difference was statistically significant.

When the adipocytes were simultaneously transfected by 100 *μ*mol/L si-KLF7 and stimulated with 100 *μ*mol/L PA for 48 h, the mRNA expression levels of the inflammatory factors p65 and IL-6 were significantly lower than the PA-siNC group (*P* < 0.05). The protein expressions of KLF7, pp65, and IL-6 were also significantly lower compared with those of the PA-siNC group (Figures 5(d) and 5(e)).

### 3.5. KLF7 Has a Transcriptional Activation Effect on IL-6

**Figure 6:**
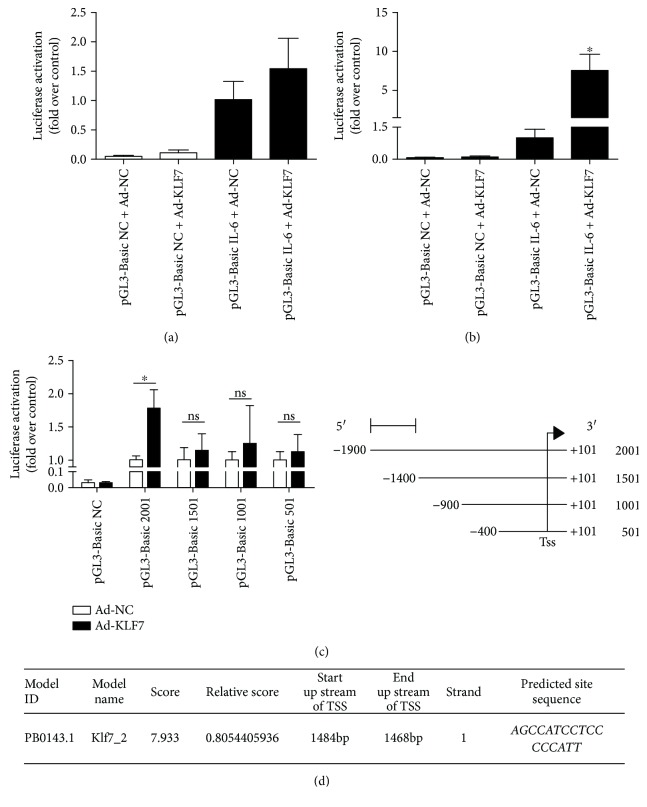
The transcriptional activation of IL-6 by KLF7, after the KLF7 overexpression plasmid and the IL-6 promoter luciferase plasmid were cotransfected into 293T cells: (a) 24 h and (b) 48 h. Activity of KLF7 on the truncated body of the IL-6 promoter region (c). The JASPAR database software predicted that KLF7 and the IL-6 promoter region are possible binding sites (d). The data for each group is derived from three replicates of experimental data. One-way ANOVA (a, c), Kruskal-Wallis *H* test (b), Ad-NC 2001: Ad-KLF7 2001, ^∗^*P* < 0.05; ns: no significant difference; the difference was statistically significant.

The human IL-6 promoter luciferase plasmid pGL-3-Basic IL-6 and the KLF7 overexpression plasmid were cotransfected to 293T cells, and the transcriptional activation was detected. The luciferase activity was significantly higher than in the control group after KLF7 overexpression and pGL-3-Basic IL-6 luciferase plasmid transfection for 48 h (*P* < 0.05; Figure 6(b)), which indicates that KLF7 has a transcriptional activation effect on IL-6.

To clarify the KLF7 and *IL-6* promoter-specific binding sites, the truncated luciferase reporter plasmid (1501 bp, 1001 bp, and 501 bp) of the core segment of the human *IL-6* promoter (length 2001 bp) was further constructed. Then we separately take the above truncated body cotransfected with the KLF7 overexpression plasmid into 293T cells. The results showed that the luciferase activity was significantly higher than in the control group after the KLF7 overexpression and pGL-3-Basic IL-6 luciferase plasmid transfection for 48 h, and the luciferase activity of the truncated 1501 bp had no significant difference with that of the control group (*P* < 0.05; Figure 6(c)). According to the JASPAR database, KLF7 could be bound to the *AGCCATCCTCCCCCATT* sequence of 1468–1484 bp upstream of the transcription initiation site of IL-6 (Figure 6(d)). Overall, the above results suggest that within the range of 1400–1900 bp upstream of the 5′ transcription start site of the *IL-6* promoter region, there is a target site that effectively binds to KLF7.

## 4. Discussion

Obesity is an important risk factor for the development of metabolic syndrome and induced inflammation, which in turn leads to insulin resistance. It is the key factor to the occurrence of metabolic syndrome [3, 4]. Adipose tissue is considered as the site of systemic inflammatory response during obesity, and inflammation of adipocytes is closely related to the accumulation of high levels of free fatty acids in the body [2]. It is reported in the research that a high-level FFA can activate the TLR4/nuclear factor-*κ*B (NF-*κ*B) signaling pathway and induce the expression and release of inflammatory factors, such as TNF-*α* and IL-6, leading to immune/inflammatory reaction [1, 10, 24]. Interleukin-6 (IL-6) acts as a pleiotropic cytokine, regulates many important physiological functions, and participates in many pathological damages. IL-6 plays an important role in the development of many clinical diseases. KLF7 plays an important regulatory role in lipid and glucose metabolism. However, there are few reports on the regulation effect of KLF7 in adipose tissue or adipocyte inflammatory response. Kawamura et al. found that the overexpression of KLF7 could upregulate IL-6 expression in human preadipocytes [19]. Above all, previous studies have shown that KLF7 may closely relate to IL-6 overexpression induced by high levels of free fatty acids in obesity. In our present study, we want to investigate the role of KLF7 in the process of FFA-induced IL-6 overexpression and to elucidate its possible mechanism in adipocytes.

Our previous experimental results suggest that the mRNA and protein expression levels of KLF7, the subunit p65 of NF-*κ*B, and the downstream inflammatory cytokine IL-6 in visceral adipose tissue were significantly higher in obese, compared with normal weight, individuals. Moreover, a significant positive correlation was found between KLF7 and IL-6 expression in visceral adipose tissue of obese individuals [22]. These results indicated that KLF7 might be involved in the obesity-induced inflammatory process. However, the specific molecular mechanism has not been reported in the literature. In the present study, the results showed that high concentrations of free fatty acids could promote the expressions of KLF7, p65, pp65, and inflammatory cytokines, such as IL-6, IL-1*β*, and MCP-1, in adipocytes, and IL-6 is rather obvious. In addition, the mRNA and protein expressions of p65, pp65, and IL-6 were significantly increased after upregulating the KLF7 expression in adipocytes. Meanwhile, the mRNA and protein expressions of pp65 and IL-6 were significantly decreased after downregulation of the KLF7 expression. When the adipocytes were simultaneously transfected with si-KLF7 and stimulated with PA, the IL-6 overexpression induced by high levels of FFA was significantly inhibited. The above results suggest that free fatty acids can promote the expression of IL-6 by upregulating KLF7 in adipocytes. However, the specific molecular mechanism by which FFA promotes high expression of KLF7 in adipocytes is still unclear.

Toll-like receptors (TLRs) play an important role in the innate immune response inflammatory signaling pathway. FFA has been reported to activate the myeloid differentiation factor 88- (MyD88-) dependent pathway by directly binding to TLR4 on the surface of adipocytes, after which it activates NF-*κ*B via MyD88 and interleukin-1 receptor-associated kinase (IRAK) to make p65 into the nucleus and transcriptionally activates the downstream inflammatory factors IL-6 and TNF-*α*, and then triggering the immune inflammatory reaction [5–13]. Is there a transcription factor involved in the activation of IL-6 other than p65 in the FFA/TLR4/IL-6 signaling pathway? Our previous experimental results suggest a significant positive correlation between the TLR4 and KLF7 expressions in visceral adipose tissue of obese individuals. The expressions of TLR4 and KLF7 in visceral adipose tissue were higher than those in the normal control group [22]. The present study also confirms that high levels of FFA in adipocytes activate the TLR4/NF-*κ*B/IL-6 inflammatory signaling pathway. Under the stimulation of high concentration of FFA, the expression levels of TLR4 and KLF7 in adipocytes were significantly increased. Furthermore, after upregulation and downregulation of TLR4 in adipocytes, the expression level of KLF7 was found to be increased and decreased, respectively. The experimental results show that TLR4, as an upstream factor, can positively regulate the expression of KLF7. The above results in the study suggest that high levels of FFA in obesity may be involved in the regulation of the IL-6 inflammatory factor through the possible upregulation of KLF7 by activating of the membrane receptor TLR4.

On the contrary, Chen et al.'s study reported that human obese individuals have lower KLF7 expression [23]. Our previous experimental results suggest a significant positive correlation between the TLR4 and KLF7 expressions in visceral adipose tissue of obese individuals. The expressions of TLR4 and KLF7 in visceral adipose tissue were higher than those in the normal control group [22]. The normal control group (NC, 18.0 kg/m^2^ ≤ BMI ≤ 23.9 kg/m^2^, *n* = 50) and the obesity group (OB, BMI ≥ 28 kg/m^2^, *n* = 45). When using PA to stimulate adipocytes, our results are consistent with previous experimental results. We consider that the different results compared with Chen et al.'s are caused by the differences of the number of samples. In addition, in our study, the visceral adipose tissue samples came from Uighur individuals, who have a different genetic background and lifestyle. These reasons may also be an interesting potential factor leading to the differences in results.

The current results suggest that KLF7 can promote the phosphorylation of the NF-*κ*B subunit p65. How does KLF7 regulate p65 phosphorylation? It has been reported in the study that the protein kinase C *ζ* (PKC*ζ*) can promote I*κ*B phosphorylation by activating the I*κ*B kinase. Moreover, PKC*ζ* phosphorylates serine at positions 311 and 536 of the NF-*κ*B subunit p65, eventually leading to NF-*κ*B activation [25]. The latest reports indicate that members of the KLF family act as cotranscriptional factors of NF-*κ*B, which in turn regulates its downstream target genes. KLF4 binds to p65 and interacts with the promoter region of iNOS to promote its expression [26]. As a coregulator of NF-*κ*B, KLF6 regulates the expression of downstream inflammatory cytokines, such as MCP-1 and IL-8 [27]. Based on the above studies, we suspect that KLF7 may phosphorylate I*κ*B and p65 by promoting the PKC*ζ* expression, ultimately activating NF-*κ*B. Simultaneously, we suspect that KLF7 binds to phosphorylated p65 in the nucleus and coregulates IL-6 expression as a cotranscriptional factor. Nevertheless, further experiments are needed to verify the above assumptions.

The present results suggest that there is a significantly positive correlation between the KLF7 and the IL-6 expression. As a transcription factor, can KLF7 directly transactivate IL-6? It has been reported in the study that the transcription factor KLF7 tends to bind to GC-rich sequences in the genome. In addition, gel retardation studies have shown that KLF7 binds to the *CACCC* motif of the target gene [28]. A search of the NCBI database indicates that there is a potential *CACCC* motif binding to KLF7 in the *IL-6* gene promoter region. Therefore, we constructed the luciferase plasmid of the *IL-6* promoter region and detected the transcriptional activation of IL-6 by KLF7 with the use of a luciferase reporter assay. The results indicate that the region of 1400–1900 bp upstream of the transcription initiation site of the *IL-6* gene promoter may contain a segment that binds to KLF7 and plays a key role in transcriptional activation. This is inconsistent with the possible target binding sites for KLF7 and *IL-6* promoter regions predicted by the JASPAR database to be *AGCCATCCTCCCCCATT* sequence positions from 1468 to 1484 upstream of the 5′ transcriptional start site.

Our results do not confirm that a single factor of KLF7 activates IL-6 in the nucleus. On the one hand, the results suggest that KLF7 may directly transcriptionally activate IL-6. On the other hand, Krüppel-like factor 6 is a coactivator of NF-*κ*B that mediates p65-dependent transcription of selected downstream genes [27]. KLF7 is likely to activate IL-6 with p65-dependent transcription. Alternatively, KLF7 indirectly activates IL-6 through unknown factors. Further study is needed to confirm whether KLF7 directly binds to the abovementioned segment of *IL-6* and plays a role in the transcriptional activation. At the same time, further study is needed to clarify whether KLF7 is a coactivator of p65 to activate IL-6.

**Figure 7:**
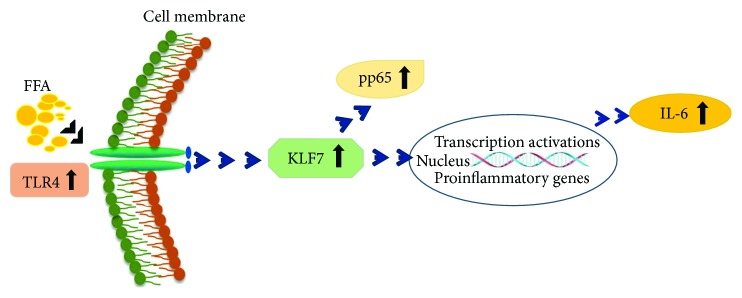
Schematic diagram: KLF7 plays an important role in the activation of FFA/TLR4/IL-6 inflammatory signaling pathways in obesity.

In summary, high concentrations of free fatty acids play an important role in the activation of TLR4/NF-*κ*B/IL-6 inflammatory signaling pathways by promoting the expression of KLF7 in obesity (Figure 7). Exploring the specific molecular mechanism of KLF7 involvement in this process would provide a new target for the treatment of obesity, inflammation, and related metabolic diseases.

## Figures and Tables

**Table 1:** Small interfering RNA list.

Target gene	siRNA sequence
*Mouse-KLF7* (si-KLF7) 5′–3′	GCCAACCAGCUCUUCUCUATT
	UAGAGAAGAGCUGGUUGGCTT
*Mouse-TLR4* (si-TLR4) 5′–3′	GGACAGCUUAUAACCUUAATT
	UUAAGGUUAUAAGCUGUCCTT
Negative control (si-NC) 5′–3′	UUCUCCGAACGUGUCACGUTT
	ACGUGACACGUUCGGAGAATT

siRNA: small interfering RNA.

**Table 2:** Primer list.

Target gene	Primer sequence
*Mouse-KLF7–F*	TCCACGACACCGGCTACTT
*Mouse-KLF7–R*	GGGAGCAGCAAGGGGTCTA
*Mouse-IL-6–F*	GCTACCAAACTGGATATAATCAGGA
*Mouse-IL-6–R*	CCAGGTAGCTATGGTACTCCAGAA
*Mouse-IL-1β–F*	ATGAAGTTCCTCTCTGCAAGAGACT
*Mouse-IL-1β–R*	CACTAGGTTTGCCGAGTAGATCTC
*Mouse-MCP-1–F*	ATTGGGATCATCTTGCTGGT
*Mouse-MCP-1–R*	CCTGCTGTTCACAGTTGCC
*Mouse-p65–F*	AGGCTTCTGGGCCTTATGTG
*Mouse-p65–R*	TGCTTCTCTCGCCAGGAATAC
*Mouse-TLR4–F*	TGGCATGGCTTACACCACC
*Mouse-TLR4–R*	GAGGCCAATTTTGTCTCCACA
*Mouse-β-actin–F*	CATTGCTGACAGGATGCAGA
*Mouse-β-actin–R*	CTGATCCACATCTGCTGGAA

Primers of RNA used in qRT-PCR.

## Data Availability

The qRT-PCR data used to support the findings of this study are available from the corresponding author upon request.
